# Molecular Targeted Therapy for the Bone Loss Secondary to Pyogenic Spondylodiscitis Using Medications for Osteoporosis: A Literature Review

**DOI:** 10.3390/ijms22094453

**Published:** 2021-04-24

**Authors:** Takashi Ohnishi, Yuki Ogawa, Kota Suda, Miki Komatsu, Satoko Matsumoto Harmon, Mitsuru Asukai, Masahiko Takahata, Norimasa Iwasaki, Akio Minami

**Affiliations:** 1 Department of Orthopaedic Surgery, Hokkaido Spinal Cord Injury Center, Bibai 072-0015, Japan; snow.stream828@gmail.com (Y.O.); sudako@sf6.so-net.ne.jp (K.S.); mikiorthopaeee@yahoo.co.jp (M.K.); satoko.og.hanna@gmail.com (S.M.H.); mitsuruasukai@gmail.com (M.A.); a-minami@hokkaidoh-s.johas.go.jp (A.M.); 2 Department of Orthopaedic Surgery, Faculty of Medicine and Graduate School of Medicine, Hokkaido University, Sapporo 060-8638, Japan; takamasa@med.hokudai.ac.jp (M.T.); niwasaki38@yahoo.co.jp (N.I.)

**Keywords:** romosozumab, teriparatide, denosumab, bisphosphonate, pyogenic spondylodiscitis, osteolysis, osteoblastogenesis, osteoclastogenesis, Wnt

## Abstract

Pyogenic spondylodiscitis can cause severe osteolytic and destructive lesions in the spine. Elderly or immunocompromised individuals are particularly susceptible to infectious diseases; specifically, infections in the spine can impair the ability of the spine to support the trunk, causing patients to be bedridden, which can also severely affect the physical condition of patients. Although treatments for osteoporosis have been well studied, treatments for bone loss secondary to infection remain to be elucidated because they have pathological manifestations that are similar to but distinct from those of osteoporosis. Recently, we encountered a patient with severely osteolytic pyogenic spondylodiscitis who was treated with romosozumab and exhibited enhanced bone formation. Romosozumab stimulated canonical Wnt/β-catenin signaling, causing robust bone formation and the inhibition of bone resorption, which exceeded the bone loss secondary to infection. Bone loss due to infections involves the suppression of osteoblastogenesis by osteoblast apoptosis, which is induced by the nuclear factor-κB and mitogen-activated protein kinase pathways, and osteoclastogenesis with the receptor activator of the nuclear factor-κB ligand-receptor combination and subsequent activation of the nuclear factor of activated T cells cytoplasmic 1 and c-Fos. In this study, we review and discuss the molecular mechanisms of bone loss secondary to infection and analyze the efficacy of the medications for osteoporosis, focusing on romosozumab, teriparatide, denosumab, and bisphosphonates, in treating this pathological condition.

## 1. Introduction

Pyogenic spondylodiscitis is an infection that occurs at an intervertebral disc and the adjacent vertebrae, and it can lead to bone loss and subsequent instability of the spine. Elderly and immunocompromised people are particularly susceptible to spondylodiscitis, have a high risk of being bedridden, and experience a deterioration of their physical condition [1]. Moreover, the prevalence of spondylodiscitis has increased over the last 20 years [1]. The prevalence of spondylodiscitis increased from 2.2/100,000 to 5.8/100,000 within 14 years in Denmark [2] and from 5.3/100,000 to 7.4/100,000 within 3 years in Japan [1,3]. Regarding the treatment, antibiotics can be effective in fighting microbes; however, they have no reparative effects on destroyed bone lesions. Notably, bone loss may have fewer treatment options than infection and tends to become problematic. A possible therapeutic measure is the administration of medications for osteoporosis. These medications modulate bone formation and/or resorption, and the effects vary by the type and characteristics of the medication. However, currently, there is no consensus on which agent is appropriate for these patients.

**Figure 1:**
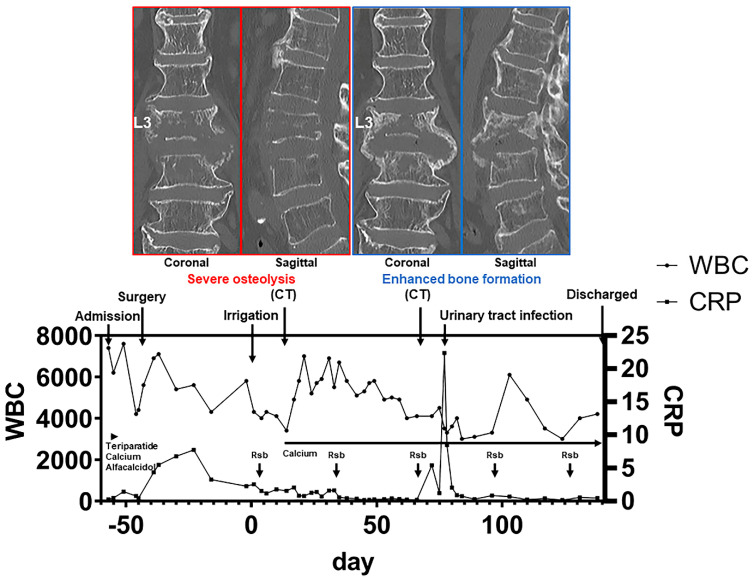
CT images show the severely destroyed osteolytic lesions due to infection and enhanced bone formation at the same site after the administration of romosozumab. The events during hospitalization are illustrated together. Rsb indicates romosozumab subcutaneous injection.

Recently, we managed the case of a patient with postoperative pyogenic osteolytic spondylodiscitis who was treated with romosozumab, a monoclonal antibody against sclerostin that stimulates canonical Wnt/β-catenin signaling and exhibited enhanced bone formation following treatment. Briefly, the elderly woman with diffuse idiopathic skeletal hyperostosis and untreated severe osteoporosis had a lumbar vertebral fracture at the L3 level. She was administered teriparatide, calcium, and alfacalcidol, but these medications were stopped after a short period because exanthema occurred. She underwent surgical fixation of the spine from the T12 to the L5 level using a percutaneous pedicle screw system but developed a surgical-site infection in the L3 vertebral body and L2/3 and L3/4 intervertebral discs six weeks after the surgery. Severe osteolysis was evident at the surgical site on the CT images (Figure 1). We started the administration of romosozumab immediately after the implant removal and thorough irrigation and continued it in parallel with the treatment of infection using antibiotics. Although serological inflammation persisted for six weeks after surgery, the CT images revealed robust bone formation in the destroyed bone lesions at the L3 vertebra and bony spur bridging at the L2/3 and L3/4 disc levels at two months after operation (Figure 1).

Although our treatment was successful in this case, a decision regarding treatment for bone loss secondary to pyogenic spondylodiscitis is still difficult and controversial. Therefore, we review the molecular signaling pathways involved in bone loss secondary to infection and discuss the potential role of the currently available osteoporosis medications, including romosozumab, teriparatide, denosumab, and bisphosphonates (BPs), in the treatment of this intractable pathological condition.

## 2. The Peculiarity of Bone Loss Secondary to Pyogenic Spondylodiscitis

The peculiarity of the spine versus other regions regarding the locus of infection is a high propensity of patients losing ambulatory competencies. Bone loss in pyogenic spondylodiscitis patients is usually rapidly progressive and highly destructive [4]; therefore, patients tend to become bedridden and cannot recover ambulatory function without restoring the support and stability of the spine. Moreover, major bone loss is due to not only the causal bacteria but also the disuse of the spine owing to bed rest, brace immobilization, and limited sunlight exposure from long hospitalization for intravenous antibiotic administration [5]. Another factor that may lead to abrogating ambulatory competence is neurological deficits. Due to its function as a container of the spinal cord and cauda equina, the disruption of the spinal column by bone loss may result in the impairment of neurological function, which is highly relevant to the patient’s ambulatory competence. The prevalence of neurological deficits, including radiculopathy, paralysis involving the trunk, limb, bladder, and rectum, and sensational disorder, is reported to be 10–50% depending on the study, and although its incidence is less than 10%, there is a chance of their progression to permanent deficits [6,7,8,9]. Fast-acting medications for bone loss are ideal for restoring the structure and function of the spine before the general body condition and neurological function deteriorates.

## 3. Molecular Mechanisms of Bone Resorption Secondary to Infection

**Figure 2:**
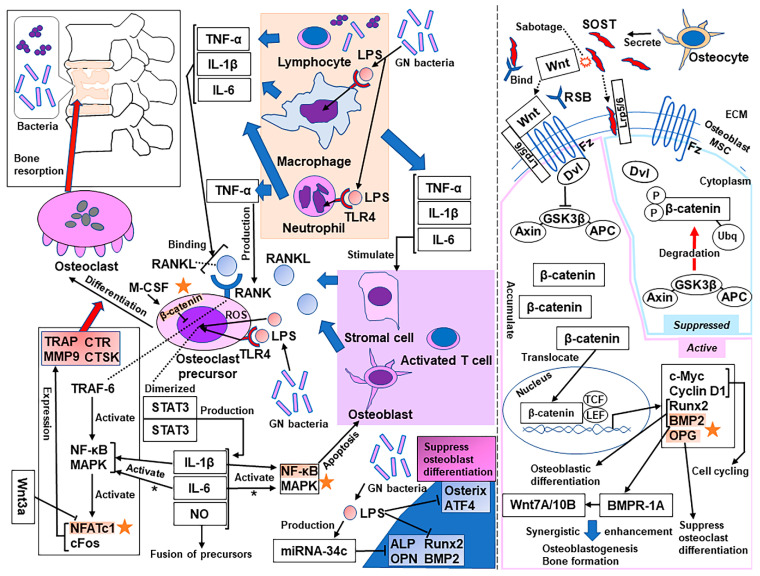
An overview of the mechanisms of bone loss secondary to infection and the effects of romosozumab to block these pathways. Orange stars indicate the sites targeted by romosozumab. * In low-level (10 ng/mL) RANKL (in contrast, IL-6 suppresses NF-kB and MAPK signaling in high-level (50 ng/mL) RANKL). BMPR-1A, BMP receptor type IA; Dvl, Disheveled; ECM, extracellular matrix; Fz, Frizzled; GN, Gram-negative; M-CSF, macrophage-colony-stimulating factor; NO, nitrogen oxide; ROS, reactive oxygen species; RSB, romosozumab; SOST, sclerostin; Ubq, ubiquitination.

Bone resorption driven by osteoclastogenesis is the primary factor responsible for bone loss secondary to pyogenic osteomyelitis. Osteoclastogenesis is initiated by proinflammatory cytokines secreted by lymphocytes and macrophages, such as tumor necrosis factor (TNF)-α, interleukin (IL)-1β, and IL-6, inducing the binding of the receptor activator of nuclear factor-κB (NF-κB) ligand (RANKL) to RANK [10]. TNF-α promotes the development of osteoclast precursors to express RANK and the development of stromal cells, osteoblasts, and activated T cells to express RANKL [10,11,12]. IL-1β also facilitates the expression of RANKL and causes osteoclast precursors to differentiate into mature osteoclasts [10,13,14]. IL-6 induces the expression of RANKL in osteoblasts and stromal cells [10,15]. Subsequently, the RANK-RANKL combination causes TNF receptor-associated factor 6 (TRAF-6) to activate NF-κB and MAPKs, followed by the activation of the transcription factors (TFs) nuclear factor of activated T cells cytoplasmic 1 (NFATc1) and c-Fos in osteoclast precursors [10,16]. These factors upregulate tartrate-resistant acid phosphatase (TRAP), matrix metalloproteinase 9 (MMP9), calcitonin receptor (CTR), and cathepsin K (CTSK), which differentiate the multinucleated osteoclasts with the assistance of macrophage-colony-stimulating factor, which stimulates the proliferation and survival of osteoclast precursors, leading to bone resorption [10,14,16]. Another important transcription factor that induces proinflammatory cytokines is signal transducer and activator of transcription (STAT) [10]. STAT3 has been demonstrated to play an important role in the production of IL-1β, IL-6, and nitrogen oxide and the subsequent differentiation of cultured preosteoclasts to mature osteoclasts induced by lipopolysaccharide (LPS) [10,17,18,19]. Although the patient in our study had an infection caused by methicillin-resistant coagulase-negative staphylococci, Gram-negative bacteria also commonly cause pyogenic spondylodiscitis. LPS is a component of the outer membrane of Gram-negative bacteria and has been shown to play important roles in bone resorption secondary to pyogenic osteomyelitis [20]. LPS activates Toll-like receptor 4 (TLR4) and stimulates various signaling pathways, including the NF-κB, MAPK, and STAT3 pathways [10,21]. The effects of LPS on bone loss include not only the stimulation of bone resorption by osteoclasts but also the inhibition of osteoblast differentiation [22,23]. LPS suppresses the expression of runt-related transcription factor (Runx) 2, osterix, and activating transcription factor 4 (ATF4), consequently inhibiting the differentiation of cultured osteoblasts [22]. The NF-κB and MAPK pathways also contribute to osteoblast apoptosis [23]. Furthermore, noncoding microRNAs (miRNAs) are single-stranded RNAs that have 18–24 nucleotides and a hairpin structure [24]. miRNAs have been confirmed to function as gene regulators posttranscriptionally [24]. miRNA-34c is upregulated by LPS stimulation, and its overexpression has been demonstrated to suppress the expression of osteogenic gene markers, such as alkaline phosphatase, Runx2, osteopontin (OPN), and bone morphogenetic protein (BMP) 2 [23] (see Figure 2).

## 4. Osteoporosis Medications as a Treatment for Bone Loss Secondary to Infection

Some medications used for osteoporosis are considered potential therapeutics for bone loss secondary to infection because these medications are used to treat bone loss in other pathological conditions. Increased osteoclastic activity is evident in many osteopenic disorders, including Paget’s disease, lytic bone metastases, and rheumatoid arthritis, causing increased bone resorption and destruction [25]. Osteoporosis medications comprise anabolic drugs and antiresorptive drugs.

Major options for anabolic medications are teriparatide and romosozumab. Teriparatide has been reported to be effective in the treatment of medication-related osteonecrosis of the jaw (MRONJ) [26], which has a pathological manifestation similar to that of pyogenic osteomyelitis. The pathological manifestation of MRONJ is considered to involve the antiresorptive effect of BPs or denosumab, a reduction in macrophages, and an increase in monocytes by the effect of BPs, local bacterial infection, inflammation, and necrosis [26,27]. However, there is increasing evidence that infection can be a histological hallmark of MRONJ [28,29,30,31,32]. Bacteria form biofilms to protect themselves from the host immune system and antibiotics [32]. Teriparatide has been suggested to promote osseous wound healing in cases of MRONJ [26,33]. When considering the use of teriparatide in the treatment of bone loss secondary to an infection, one concern is that teriparatide is known to promote bone resorption concomitant with bone formation, as shown by an elevation in the levels of serum N-telopeptide of type I collagen or collagen cross-linked C-telopeptide, although an anabolic window exists in cases of usual osteoporosis [33,34,35,36]. Romosozumab, a monoclonal antibody against sclerostin, seems to be a better choice because it has been reported to increase bone formation and decrease bone resorption [37,38,39,40,41].

The main players among antiresorptive drugs are a group of BPs and denosumab. Although the area of bone loss is systemic in the established stage, rheumatoid arthritis (RA) causes local periarticular bone loss in the preclinical stage [42], which leads to mild but similar features to those of bone infection. Many inflammatory cytokines involved in RA have osteoclastogenic effects [43], and they commonly accompany the bone loss secondary to an infection, as described in the previous section. In patients with RA, bone loss is treated with BPs or denosumab, which has been shown to significantly increase the bone mass index at the lumbar spine and hip [42]. These drugs have also been used in bone loss secondary to tumorigenesis, such as cancer metastasis or osteolytic primary bone tumors, for example, a giant-cell tumor of bone [44,45,46]. Although these medications reduce bone resorption, they are also known to suppress bone formation, resulting in decreased bone turnover [32]. Based on these facts, we will further introduce the currently available evidence for the individual drugs.

### 4.1. Anabolic Drug: Romosozumab

Romosozumab is a humanized monoclonal sclerostin antibody that sabotages sclerostin and inhibits the suppression of canonical Wnt/β-catenin (cWnt) signaling, resulting in osteoblastogenesis, bone formation, and the suppression of osteoclastogenesis [47,48,49]. Romosozumab prevents sclerostin from binding to the low-density lipoprotein receptor-related protein (LRP)-5 or LRP-6, the receptors of Wnt ligands, increasing bone formation and decreasing bone resorption [50]. The Wnt ligands, without restriction by sclerostin, bind to a specific Frizzled family receptor and an LRP-5/6 coreceptor on the surface of the osteoblasts and activate Disheveled, which prevents glycogen synthase kinase 3β (GSK3β) from phosphorylating β-catenin, forming a complex with adenomatous polyposis coli (APC) and Axin [51]. Cytoplasmic β-catenin accumulates, escapes ubiquitination and degradation by proteasomes, translocates into the nucleus, and binds to the transcription factor T cell factor/lymphoid enhancer factor (TCF/LEF), upregulating the target gene expression [51,52]. cWnt signals are involved in the self-renewal, proliferation, and epithelial-mesenchymal transition of target cells [53] and affect fibroblast growth factor, Notch, and transforming growth factor-β signals [49,54,55,56]. Specifically, in bone, cWnt signals promote the Runx2-dependent osteoblastic differentiation of mesenchymal stem cells or progenitor cells and have an anabolic effect [49,57,58]. The activation of cWnt signaling induced by Wnt3a or the overexpression of β-catenin/TCF4 also elevates BMP2 promoter activity and its mRNA levels [59]. This mechanism has been validated by confirming the existence of TCF/LEF response elements in the BMP2 promoter region using a site-directed mutagenesis approach [59]. Furthermore, cWnt signaling upregulates BMP2 and then upregulates Wnt7A/10B through BMP receptor type IA, resulting in the synergistic enhancement of osteoblastogenesis and bone formation [47,49,59]. Moreover, cWnt signaling causes antiresorptive effects by suppressing osteoclast differentiation at the precursor level [52]. cWnt signaling in osteoblasts elevates the expression of osteoprotegerin (OPG) and suppresses osteoclast differentiation [60,61]. In contrast, osteoclast precursor-specific β-catenin knockout mice showed enhanced osteoclast differentiation and osteopenia [52]. This result demonstrated that cWnt signaling also suppresses osteoclastogenesis at the precursor level in an OPG-independent manner [52]. In another study, early treatment with Wnt3a inactivated the crucial transcription factor NFATc1 in osteoclast precursors [60]. Although Wnt3a is known to activate cWnt signaling, that study revealed the involvement of noncanonical Wnt signaling in the Wnt3a inactivation of NFATc1 by demonstrating that the deletion of β-catenin does not block this inactivation [60]. In brief, Wnt3a activates cyclic adenosine monophosphate (cAMP)/protein kinase A (PKA) signaling and suppresses osteoclast differentiation by phosphorylating NFATc1 and suppressing its nuclear localization [60]; thus, romosozumab may also affect the noncanonical signals induced by Wnt3a. In addition, PKA has also been shown to negatively regulate NF-kB nuclear localization; therefore, romosozumab may contribute to inhibiting apoptosis of osteoblasts (see Figure 2).

In our experience with this patient, the effects of romosozumab against bone loss secondary to infection presumably addressed the osteoclastogenesis with regard to the parts driven by NFATc1 activation, the role of OPG, and the role of β-catenin in suppressing osteoclast differentiation. However, romosozumab was also effective in modulating osteoblastogenesis and consequent bone formation in concert with BMP2 signaling, as well as by hindering the osteoblast apoptosis induced by the NF-κB pathways (see Figure 2). The patient exhibited enhanced bone formation at a severely osteolytic infection site as early as two months after implant removal and thorough irrigation. Importantly, serological inflammation was present for six weeks after the surgery, meaning that romosozumab strongly promoted bone formation, even in the presence of osteoclastogenesis and the suppression of osteoblastogenesis induced by infection. However, there is a lack of evidence to explicitly demonstrate the efficacy and possible disadvantages of romosozumab, and well-designed studies are necessary.

### 4.2. Anabolic Drug: Teriparatide

Teriparatide is a recombinant human parathyroid hormone (PTH) that contains the 1st to 34th amino acids of the N-terminus of intact PTH [62]. When PTH was administered at a low dose intermittently, it had anabolic effects on the skeleton [35]. Most PTH signals are transmitted through the PTH-1 receptor, a G protein-coupled protein, activated by peptide sequences in the N-terminal region [35]. PTH regulates osteoblast function through the activation of the cAMP-dependent PKA and calcium-dependent protein kinase C signaling pathways [35,63]. The other mechanisms that are involved are the MAP kinase and phospholipase A and D pathways [35]. In addition, the expression of the Wnt antagonist sclerostin was downregulated by chronic and excess administration of PTH, which explains the anabolic effects of PTH, although intermittent daily administration failed to suppress sclerostin [64]. PTH acts directly on osteoblast lineage cells through an antiapoptotic action by Runx2 in intermittent administration and indirectly affects those cells through growth factors, such as IGF-I, and growth factor antagonists, such as sclerostin [35,63,64]. IGF-I synthesized in osteoblasts results in potent anabolic effects in concert with the direct effects of PTH on cancellous bone [35].

Despite the accumulation of knowledge about teriparatide, there are still a limited number of studies reporting the effects of teriparatide on bone loss secondary to infection. There are a few case reports of the effect of teriparatide on septic arthritis with and without arthroplasty, pyogenic osteomyelitis and nonunion of the tibia, and pyogenic spondylitis, all of which concluded that teriparatide was effective on this pathological condition without the aggravation of infection [65,66,67,68]. Although not simulating the exact situation of pyogenic osteomyelitis, regarding the molecular mechanism of how teriparatide works on the bone when the host is undergoing sepsis, Terashima et al. studied its effect using cecal ligation and puncture-treated mice [69]. Previous studies have demonstrated that IL-7 stimulates the proliferation and activation of lymphoid cells and improves host survival in sepsis [69,70,71]. In the study of Terashima et al., sepsis resulted in a rapid ablation of osteoblasts and subsequent reduction in common lymphoid progenitors via the effects of granulocyte colony-stimulating factor, leading to lymphopenia-associated immunodeficiency, but sepsis caused no change in osteoclasts [69]. Interestingly, the use of teriparatide not only stimulated the proliferation of osteoblasts but also improved the lymphopenia induced by sepsis through an increase in IL-7 [69]. The occurrence of consequent augmentation of T and B cells was not limited to peripheral blood but was also evident in the bone marrow [69]. Considering these findings, teriparatide may positively affect bone loss secondary to pyogenic myelitis by bone formation, boosting the proliferation of osteoblasts and strengthening the immune system by increasing the number of lymphocytes through IL-7 signaling. However, further studies are needed to rule out adverse side effects, such as paradoxical bone resorption or hypercalcemia, when using teriparatide in an acute phase of bone infection.

### 4.3. Antiresorptive Drug: Denosumab

Denosumab is a human monoclonal antibody against RANKL that inhibits the differentiation of osteoclast precursors into mature osteoclasts [72]. However, because its receptor, RANK, is also expressed by monocytes/macrophages and dendritic cells, and RANKL, RANK, and OPG deficiency in murine models underscores the importance of this pathway in the development and maturation of the immune system [25,73,74,75,76], a concern arose regarding potential infectious events when RANKL was inhibited [72]. Initially, the results from a clinical trial, fracture reduction evaluation of denosumab in osteoporosis every 6 months (FREEDOM), showed that there was no increase in the risk of serious infections [77]. However, a more recent meta-analysis concluded that there was a significant increase in the risk of serious infections in osteoporosis in females or osteopenia patients treated with denosumab [77,78]. This result was supported by newer data from the FREEDOM trial showing concordant results, although the risk increase fell into borderline significance when nonmetastatic breast cancer was excluded [78,79]. Watts et al. also evaluated infectious events in the FREEDOM trial, reporting a more frequent incidence of erysipelas and cellulitis and infections in other regions, such as the gastrointestinal system, renal and urinary system, ear, and endocardium [80]. However, the number of each event was small, and there was no relationship between infectious events and the timing of the administration or duration of denosumab [80], which leads to skepticism regarding causality between the administration of denosumab and the occurrence of infectious events. In summary, denosumab might be associated with an increase in serious infections [81] based on the underlying evidence in the area of molecular science. Denosumab may, therefore, cause the deterioration of infection when used in pyogenic osteomyelitis patients, even if it was effective in suppressing osteolysis. Likewise, further studies are necessary to clarify the efficacy of denosumab in the treatment of this pathological condition.

### 4.4. Antiresorptive Drug: BPs

BPs are a group of pyrophosphate analogs that bind to hydroxyapatite on the bone surface at the sites where remodeling is active and are internalized into osteoclasts through endocytosis, leading to their apoptosis or the suppression of bone-resorbing activity [82]. BPs comprise two types of chemical structures specific to the side-chain groups: nonnitrogen-containing BPs (non-NBPs) and nitrogen-containing BPs (NBPs), which exhibit more powerful effects than the prior and are widely used [83]. Non-NBPs exert their activities by conversion into cytotoxic ATP analogs, interfering with mitochondrial function in osteoclasts and leading to apoptosis [84,85]. NBPs bind to key enzymes of the intracellular mevalonate pathway and inhibit them, preventing the prenylation and activation of small GTPases that are essential for the bone-resorbing activity and survival of osteoclasts [82,86,87,88,89]. The mevalonate pathway is responsible for the production of cholesterol and isoprenoid lipids [82,90]. Some of the isoprenoid lipids are indispensable for the prenylation and activation of GTPases [82,90,91,92]. These GTPases play an important role in regulating osteoclast morphology, cytoskeleton arrangement, membrane ruffling, trafficking, and cell survival [82,93,94].

In the context of using BPs as a treatment for bone loss secondary to pyogenic osteomyelitis, the majority of studies have highlighted the downsides of NBPs. First, NBPs have inflammatory side effects leading to osteomyelitis [95]. Deng et al. pretreated macrophages that were infected with *Porphyromonas gingivalis* and *Tannerella forsythia* with alendronate, an NBP, and demonstrated augmented production of IL-1β through caspase-1 activation [95]. Furthermore, intraperitoneal injection of NBPs induced histamine-forming enzyme histidine decarboxylase (HDC) in tissues, such as the liver, lungs, spleen, and bone marrow, through IL-1 signaling in murine models [96]. HDC is induced by NBPs, LPS, IL-1, and TNF, and histamine is an inflammatory mediator and a regulator of immune responses, including Th1/Th2 balance and hematopoiesis [97]. Pretreatment with alendronate augmented LPS-stimulated IL-1 production and HDC induction; conversely, pretreatment with LPS augmented alendronate-induced HDC elevation [96]. Moreover, macrophages activate human γδ T cells when treated with NBPs, and it is suggested that macrophages present NBPs to γδ T cells [96,98]. Another study reported that NBPs stimulated human γδ T cells to release TNFα and/or interferon-γ [99] through the inhibition of the mevalonate pathway [100].

Second, NBPs downregulated TLR ligand-induced monocyte chemoattractant protein-1 (MCP-1) and macrophage inflammatory protein-1α (MIP-1α) production in the macrophage-like cell line J774.1 via Smad3 activation [101]. The chemokine MCP-1 facilitates osteoclast differentiation [102], and MIP-1α stimulates osteoclasts [103]; therefore, a reduction in these chemokines might inhibit the normal activation and migration of osteoclasts and cause osteonecrosis, leading to the formation of sequestra [101].

Third, the existence of NBPs on the surface of the bone can significantly increase the number of bacteria attached to the bone [104]. When pamidronate was used to coat a hydroxyapatite (HA) material, the number of adherent bacteria was 60-fold greater than that when the HA was uncoated; therefore, NBPs presumably increase the bacterial load at the infection site and exacerbate the infection [104].

**Figure 3:**
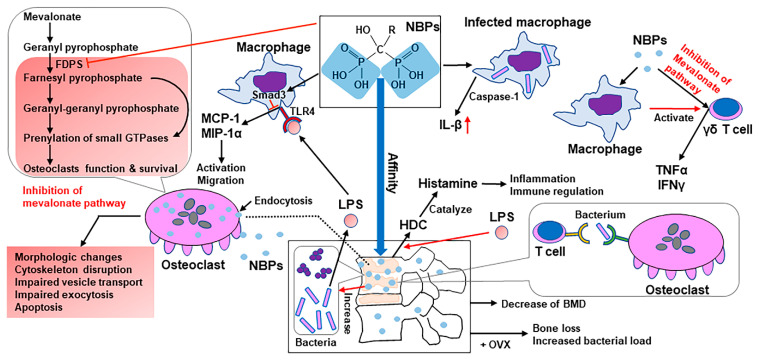
The molecular mechanisms of the effects of nitrogen-containing bisphosphonates (NBPs) on pyogenic osteomyelitis. BMD, bone mass density; FDPS, farnesyl diphosphate synthase; HDC, histamine-forming enzyme histidine decarboxylase; IFN-γ, interferon-γ; IL-1β, interleukin-1β; LPS, lipopolysaccharide; MCP-1, monocyte chemoattractant protein-1; MIP-1α, macrophage inflammatory protein-1α; OVX, ovariectomy; TLR4, Toll-like receptor 4; TNFα, tumor necrosis factor α.

Fourth, the insufficient efficacy of BPs on bone loss secondary to pyogenic osteomyelitis can also be pointed out. Kim et al. retrospectively analyzed the efficacy of BPs in pyogenic vertebral osteomyelitis patients, subgrouping as follows: group A, patients who received BPs within 6 weeks after diagnosis; group B, patients who received BPs between 6 weeks and 3 months after diagnosis; and group C, patients who received no treatment for osteoporosis [5]. Although the hazard ratios for the recurrence of infection were not significantly different among the three groups, bone mineral densities measured by dual-energy X-ray absorptiometry decreased by 0.7% in group A and 1.7% in group B at the lumbar spine one year after the diagnosis [5]. In another study, the administration of an NBP aggravated the infection. This study characterized the bone changes resulting from *Staphylococcus epidermidis* infection in a rodent orthopedic device-related infection model and further evaluated whether ovariectomy (OVX) or BP treatment influenced the infection [105]. As a result, treatment with zoledronic acid did not have bone-protective effects on OVX-infected animals; moreover, it significantly increased the bacterial load, suggesting that osteoclasts might be important in the control of the infection [105]. Supporting this theory, there is a study reporting osteoclasts as immune-competent cells that can internalize and present bacterial antigens to T cells [106]. Several studies have reported that NBPs cause patients to be more susceptible to infection [107,108]. Although one study reported a protective role of zoledronic acid on healing tooth extraction wounds and bone loss in a mouse model of pyogenic osteomyelitis of the jaw [109], this may be limited to the oral region environment. The majority of the studies reported the disadvantages of NBPs in treating bone loss secondary to pyogenic osteomyelitis (see Figure 3).

Evidence for each drug regarding the therapeutic effects on pyogenic osteomyelitis is recapitulated in Table 1.

## 5. Conclusions

Considering the underlying characteristics of bone loss in pyogenic spondylodiscitis and the relatively older population of patients with this condition, the use of anabolic medications may be desirable in these patients, given the potential advantages reported in previous studies. Further accumulation of evidence will help in the treatment of patients with this intractable disease, which is increasing in prevalence and impacts society.

## Figures and Tables

**Table 1:** Advantages and disadvantages of romosozumab, teriparatide, denosumab, and bisphosphonates (BPs) in the treatment for pyogenic osteomyelitis.

Drugs	Advantages	Disadvantages
Romosozumab *	Suppresses osteoclastogenesis by phosphorylating NFATc1, elevating the expression of OPG, and accumulation of β-catenin in osteoclasts Enhances osteoblastogenesis via augmenting BMP2 signaling and hindering osteoblast apoptosis induced by the NF-κB pathways	Unknown with regard to the areas of the bone and infection
Teriparatide	Stimulates the proliferation of osteoblasts Stimulates the proliferation and activation of lymphoid cells via upregulating IL-7 in the bone marrow	Unknown with regard to the areas of the bone and infection, but may need precaution to paradoxical bone resorption or hypercalcemia
Denosumab	Inhibits osteoclastogenesis by inhibiting RANKL	A concern of aggravating the infection
NBPs	Impair function and survival of osteoclasts One study reported that zoledronic acid had protective effects on bone loss secondary to pyogenic osteomyelitis of the jaw	Augment the production of IL-1β in macrophages Upregulate histamine production via HDC induction with and without the LPS existence in the bone marrow Affect macrophages to activate γδ T cells Stimulate γδ T cells to release TNFα and/or IFNγ Down-regulate TLR ligand-induced MCP-1 and MIP-1α production in macrophages Increase bacterial loads at the infection site Insufficient efficacy on the bone loss

* Romosozumab lacks evidence from previous studies; therefore, we discussed based on the known efficacy of romosozumab. BMP2, bone morphogenetic protein 2; HDC, histamine-forming enzyme histidine decarboxylase; IFNγ, interferon-γ; IL, interleukin; LPS, lipopolysaccharide; MCP-1, monocyte chemoattractant protein-1; MIP-1α, macrophage inflammatory protein-1α; NBPs, nitrogen-containing BPs; NFATc1, nuclear factor of activated T cells cytoplasmic 1; NF-κB, nuclear factor-κB; OPG, osteoprotegerin; RANKL, receptor activator of NF-κB ligand; TLR, Toll-like receptor; TNFα, tumor necrosis factor α.

## References

[1] Pola E., Taccari F., Autore G., Giovannenze F., Pambianco V., Cauda R., Maccauro G., Fantoni M. (2018). Multidisciplinary management of pyogenic spondylodiscitis: Epidemiological and clinical features, prognostic factors and long-term outcomes in 207 patients. Eur. Spine J..

[2] Kehrer M., Pedersen C., Jensen T.G., Lassen A.T. (2014). Increasing incidence of pyogenic spondylodiscitis: A 14-year population-based study. J. Infect..

[3] Akiyama T., Chikuda H., Yasunaga H., Horiguchi H., Fushimi K., Saita K. (2013). Incidence and risk factors for mortality of vertebral osteomyelitis: A retrospective analysis using the Japanese diagnosis procedure combination database. BMJ Open.

[4] Shousha M., Boehm H. (2012). Surgical treatment of cervical spondylodiscitis: A review of 30 consecutive patients. Spine.

[5] Kim J., Jang S.B., Kim S.W., Oh J.K., Kim T.H. (2019). Clinical effect of early bisphosphonate treatment for pyogenic vertebral osteomyelitis with osteoporosis: An analysis by the Cox proportional hazard model. Spine J..

[6] Kwon J.W., Hyun S.J., Han S.H., Kim K.J., Jahng T.A. (2017). Pyogenic Vertebral osteomyelitis: Clinical features, diagnosis, and treatment. Korean J. Spine.

[7] Mylona E., Samarkos M., Kakalou E., Fanourgiakis P., Skoutelis A. (2009). Pyogenic vertebral osteomyelitis: A systematic review of clinical characteristics. Semin. Arthritis Rheum..

[8] Sato K., Yamada K., Yokosuka K., Yoshida T., Goto M., Matsubara T., Iwahashi S., Shimazaki T., Nagata K., Shiba N. (2019). Pyogenic spondylitis: Clinical features, diagnosis and treatment. Kurume Med. J..

[9] Lam K.S., Webb J.K. (2004). Discitis. Hosp. Med..

[10] Yang J., Tang R., Yi J., Chen Y., Li X., Yu T., Fei J. (2019). Diallyl disulfide alleviates inflammatory osteolysis by suppressing osteoclastogenesis via NF-κB-NFATc1 signal pathway. FASEB J..

[11] Komine M., Kukita A., Kukita T., Ogata Y., Hotokebuchi T., Kohashi O. (2001). Tumor necrosis factor-alpha cooperates with receptor activator of nuclear factor kappaB ligand in generation of osteoclasts in stromal cell-depleted rat bone marrow cell culture. Bone.

[12] Kitaura H., Kimura K., Ishida M., Kohara H., Yoshimatsu M., Takano-Yamamoto T. (2013). Immunological reaction in TNF-α-mediated osteoclast formation and bone resorption in vitro and in vivo. Clin. Dev. Immunol..

[13] Ruscitti P., Cipriani P., Carubbi F., Liakouli V., Zazzeroni F., di Benedetto P., Berardicurti O., Alesse E., Giacomelli R. (2015). The role of IL-1β in the bone loss during rheumatic diseases. Mediators Inflamm..

[14] Jules J., Zhang P., Ashley J.W., Wei S., Shi Z., Liu J., Michalek S.M., Feng X. (2012). Molecular basis of requirement of receptor activator of nuclear factor κB signaling for interleukin 1-mediated osteoclastogenesis. J. Biol. Chem..

[15] Yoshitake F., Itoh S., Narita H., Ishihara K., Ebisu S. (2008). Interleukin-6 directly inhibits osteoclast differentiation by suppressing receptor activator of NF-kappaB signaling pathways. J. Biol. Chem..

[16] Quan G.H., Wang H., Cao J., Zhang Y., Wu D., Peng Q., Liu N., Sun W.C. (2015). Calycosin suppresses RANKL-mediated osteoclastogenesis through inhibition of MAPKs and NF-κB. Int. J. Mol. Sci..

[17] Park H., Noh A.L., Kang J.H., Sim J.S., Lee D.S., Yim M. (2015). Peroxiredoxin II negatively regulates lipopolysaccharide-induced osteoclast formation and bone loss via JNK and STAT3. Antioxid. Redox. Signal..

[18] Kim J.H., Jin H.M., Kim K., Song I., Youn B.U., Matsuo K., Kim N. (2009). The mechanism of osteoclast differentiation induced by IL-1. J. Immunol..

[19] Feng W., Liu H., Luo T., Liu D., Du J., Sun J., Wang W., Han X., Yang K., Guo J. (2017). Combination of IL-6 and sIL-6R differentially regulate varying levels of RANKL-induced osteoclastogenesis through NF-κB, ERK and JNK signaling pathways. Sci. Rep..

[20] Guo C., Yuan L., Wang J.G., Wang F., Yang X.K., Zhang F.H., Song J.L., Ma X.Y., Cheng Q., Song G.H. (2014). Lipopolysaccharide (LPS) induces the apoptosis and inhibits osteoblast differentiation through JNK pathway in MC3T3-E1 cells. Inflammation.

[21] Lu Y.C., Yeh W.C., Ohashi P.S. (2008). LPS/TLR4 signal transduction pathway. Cytokine.

[22] Bandow K., Maeda A., Kakimoto K., Kusuyama J., Shamoto M., Ohnishi T., Matsuguchi T. (2010). Molecular mechanisms of the inhibitory effect of lipopolysaccharide (LPS) on osteoblast differentiation. Biochem. Biophys. Res. Commun..

[23] Liu J., Li D., Sun X., Wang Y., Xiao Q., Chen A. (2016). Icariine restores LPS-induced bone loss by downregulating miR-34c level. Inflammation.

[24] Zhao D., Zheng H., Greasley A., Ling F., Zhou Q., Wang B., Ni T., Topiwala I., Zhu C., Mele T. (2020). The role of miR-711 in cardiac cells in response to oxidative stress and its biogenesis: A study on H9C2 cells. Cell. Mol. Biol. Lett..

[25] Kong Y.Y., Yoshida H., Sarosi I., Tan H.L., Timms E., Capparelli C., Morony S., Oliveira-dos-Santos A.J., Van G., Itie A. (1999). OPGL is a key regulator of osteoclastogenesis, lymphocyte development and lymph-node organogenesis. Nature.

[26] Khan A.A., Morrison A., Hanley D.A., Felsenberg D., McCauley L.K., O’Ryan F., Reid I.R., Ruggiero S.L., Taguchi A., Tetradis S. (2015). Diagnosis and management of osteonecrosis of the jaw: A systematic review and international consensus. J. Bone Miner. Res..

[27] Hoefert S., Schmitz I., Weichert F., Gaspar M., Eufinger H. (2015). Macrophages and bisphosphonate-related osteonecrosis of the jaw (BRONJ): Evidence of local immunosuppression of macrophages in contrast to other infectious jaw diseases. Clin. Oral Investig..

[28] Abu-Id M.H., Warnke P.H., Gottschalk J., Springer I., Wiltfang J., Acil Y., Russo P.A., Kreusch T. (2008). “Bis-phossy jaws”—High and low risk factors for bisphosphonate-induced osteonecrosis of the jaw. J. Craniomaxillofac. Surg..

[29] Perrotta I., Cristofaro M.G., Amantea M., Russo E., de Fazio S., Zuccalà V., Conforti F., Amorosi A., Donato G., Tripepi S. (2010). Jaw osteonecrosis in patients treated with bisphosphonates: An ultrastructural study. Ultrastruct. Pathol..

[30] Sedghizadeh P.P., Kumar S.K., Gorur A., Schaudinn C., Shuler C.F., Costerton J.W. (2009). Microbial biofilms in osteomyelitis of the jaw and osteonecrosis of the jaw secondary to bisphosphonate therapy. J. Am. Dent. Assoc..

[31] Hansen T., Kunkel M., Springer E., Walter C., Weber A., Siegel E., Kirkpatrick C.J. (2007). Actinomycosis of the jaws—Histopathological study of 45 patients shows significant involvement in bisphosphonate-associated osteonecrosis and infected osteoradionecrosis. Virchows Arch..

[32] Reid I.R., Cornish J. (2011). Epidemiology and pathogenesis of osteonecrosis of the jaw. Nat. Rev. Rheumatol..

[33] Yoshiga D., Yamashita Y., Nakamichi I., Tanaka T., Yamauchi K., Yamamoto N., Nogami S., Kaneuji T., Mitsugi S., Sakurai T. (2013). Weekly teriparatide injections successfully treated advanced bisphosphonate-related osteonecrosis of the jaws. Osteoporos. Int..

[34] Tsujimoto M., Chen P., Miyauchi A., Sowa H., Krege J.H. (2011). PINP as an aid for monitoring patients treated with teriparatide. Bone.

[35] Canalis E., Giustina A., Bilezikian J.P. (2007). Mechanisms of anabolic therapies for osteoporosis. N. Engl. J. Med..

[36] Kim J.Y., Park J.H., Jung H.D., Jung Y.S. (2019). Treatment of medication-related osteonecrosis of the jaw around the dental implant with a once-weekly teriparatide: A case report and literature review. J. Oral Implantol..

[37] Chouinard L., Felx M., Mellal N., Varela A., Mann P., Jolette J., Samadfam R., Smith S.Y., Locher K., Buntich S. (2016). Carcinogenicity risk assessment of romosozumab: A review of scientific weight-of-evidence and findings in a rat lifetime pharmacology study. Regul. Toxicol. Pharmacol..

[38] Cosman F., Crittenden D.B., Ferrari S., Khan A., Lane N.E., Lippuner K., Matsumoto T., Milmont C.E., Libanati C., Grauer A. (2018). FRAME study: The foundation effect of building bone with 1 year of romosozumab leads to continued lower fracture risk after transition to denosumab. J. Bone Miner. Res..

[39] Graeff C., Campbell G.M., Peña J., Borggrefe J., Padhi D., Kaufman A., Chang S., Libanati C., Glüer C.C. (2015). Administration of romosozumab improves vertebral trabecular and cortical bone as assessed with quantitative computed tomography and finite element analysis. Bone.

[40] Lv F., Cai X., Yang W., Gao L., Chen L., Wu J., Ji L. (2020). Denosumab or romosozumab therapy and risk of cardiovascular events in patients with primary osteoporosis: Systematic review and meta-analysis. Bone.

[41] Matheny J.B., Torres A.M., Ominsky M.S., Hernandez C.J. (2017). Romosozumab treatment converts trabecular rods into trabecular plates in male cynomolgus monkeys. Calcif. Tissue Int..

[42] Raterman H.G., Lems W.F. (2019). Pharmacological management of osteoporosis in rheumatoid arthritis patients: A review of the literature and practical guide. Drugs Aging.

[43] Kitaura H., Marahleh A., Ohori F., Noguchi T., Shen W.R., Qi J., Nara Y., Pramusita A., Kinjo R., Mizoguchi I. (2020). Osteocyte-related cytokines regulate osteoclast formation and bone resorption. Int. J. Mol. Sci..

[44] Gül G., Sendur M.A., Aksoy S., Sever A.R., Altundag K. (2016). A comprehensive review of denosumab for bone metastasis in patients with solid tumors. Curr. Med. Res. Opin..

[45] Smith M.R., Saad F., Coleman R., Shore N., Fizazi K., Tombal B., Miller K., Sieber P., Karsh L., Damião R. (2012). Denosumab and bone-metastasis-free survival in men with castration-resistant prostate cancer: Results of a phase 3, randomised, placebo-controlled trial. Lancet.

[46] Mattei T.A., Ramos E., Rehman A.A., Shaw A., Patel S.R., Mendel E. (2014). Sustained long-term complete regression of a giant cell tumor of the spine after treatment with denosumab. Spine J..

[47] Chen Y., Alman B.A. (2009). Wnt pathway, an essential role in bone regeneration. J. Cell. Biochem..

[48] Ke H.Z., Richards W.G., Li X., Ominsky M.S. (2012). Sclerostin and Dickkopf-1 as therapeutic targets in bone diseases. Endocr. Rev..

[49] Katoh M., Katoh M. (2017). Molecular genetics and targeted therapy of WNT-related human diseases (review). Int. J. Mol. Med..

[50] Boyce R.W., Niu Q.T., Ominsky M.S. (2017). Kinetic reconstruction reveals time-dependent effects of romosozumab on bone formation and osteoblast function in vertebral cancellous and cortical bone in cynomolgus monkeys. Bone.

[51] Lewiecki E.M. (2014). Role of sclerostin in bone and cartilage and its potential as a therapeutic target in bone diseases. Ther. Adv. Musculoskelet. Dis..

[52] Maeda K., Kobayashi Y., Koide M., Uehara S., Okamoto M., Ishihara A., Kayama T., Saito M., Marumo K. (2019). The regulation of bone metabolism and disorders by wnt signaling. Int. J. Mol. Sci..

[53] Holland J.D., Klaus A., Garratt A.N., Birchmeier W. (2013). Wnt signaling in stem and cancer stem cells. Curr. Opin. Cell Biol..

[54] Ranganathan P., Weaver K.L., Capobianco A.J. (2011). Notch signalling in solid tumours: A little bit of everything but not all the time. Nat. Rev. Cancer.

[55] Gonzalez D.M., Medici D. (2014). Signaling mechanisms of the epithelial-mesenchymal transition. Sci. Signal..

[56] Katoh M., Nakagama H. (2014). FGF receptors: Cancer biology and therapeutics. Med. Res. Rev..

[57] Karantalis V., Hare J.M. (2015). Use of mesenchymal stem cells for therapy of cardiac disease. Circ. Res..

[58] Atashi F., Modarressi A., Pepper M.S. (2015). The role of reactive oxygen species in mesenchymal stem cell adipogenic and osteogenic differentiation: A review. Stem Cells Dev..

[59] Zhang R., Oyajobi B.O., Harris S.E., Chen D., Tsao C., Deng H.W., Zhao M. (2013). Wnt/β-catenin signaling activates bone morphogenetic protein 2 expression in osteoblasts. Bone.

[60] Weivoda M.M., Ruan M., Hachfeld C.M., Pederson L., Howe A., Davey R.A., Zajac J.D., Kobayashi Y., Williams B.O., Westendorf J.J. (2016). Wnt signaling inhibits osteoclast differentiation by activating canonical and noncanonical cAMP/PKA pathways. J. Bone Miner. Res..

[61] Fujita K., Janz S. (2007). Attenuation of WNT signaling by DKK-1 and -2 regulates BMP2-induced osteoblast differentiation and expression of OPG, RANKL and M-CSF. Mol. Cancer.

[62] Shi Z., Zhou H., Pan B., Lu L., Liu J., Kang Y., Yao X., Feng S. (2016). Effectiveness of teriparatide on fracture healing: A systematic review and meta-analysis. PLoS ONE.

[63] Dempster D.W., Cosman F., Parisien M., Shen V., Lindsay R. (1993). Anabolic actions of parathyroid hormone on bone. Endocr. Rev..

[64] Bellido T., Ali A.A., Gubrij I., Plotkin L.I., Fu Q., O’Brien C.A., Manolagas S.C., Jilka R.L. (2005). Chronic elevation of parathyroid hormone in mice reduces expression of sclerostin by osteocytes: A novel mechanism for hormonal control of osteoblastogenesis. Endocrinology.

[65] Mouyis M., Fitz-Clarence H., Manson J., Ciurtin C. (2015). Teriparatide: An unexpected adjunct for the treatment of a long-standing infected elbow prosthesis prevented arm amputation. Clin. Rheumatol..

[66] Nishikawa M., Kaneshiro S., Takami K., Owaki H., Fuji T. (2019). Bone stock reconstruction for huge bone loss using allograft-bones, bone marrow, and teriparatide in an infected total knee arthroplasty. J. Clin. Orthop. Trauma.

[67] Rollo G., Luceri F., Falzarano G., Salomone C., Bonura E.M., Popkov D., Ronga M., Pica G., Bisaccia M., Russi V. (2021). Effectiveness of teriparatide combined with the Ilizarov technique in septic tibial non-union. Med. Glas..

[68] Shinohara A., Ueno Y., Marumo K. (2014). Weekly teriparatide therapy rapidly accelerates bone healing in pyogenic spondylitis with severe osteoporosis. Asian Spine J..

[69] Terashima A., Okamoto K., Nakashima T., Akira S., Ikuta K., Takayanagi H. (2016). Sepsis-induced osteoblast ablation causes immunodeficiency. Immunity.

[70] Unsinger J., McGlynn M., Kasten K.R., Hoekzema A.S., Watanabe E., Muenzer J.T., McDonough J.S., Tschoep J., Ferguson T.A., McDunn J.E. (2010). IL-7 promotes T cell viability, trafficking, and functionality and improves survival in sepsis. J. Immunol..

[71] Venet F., Foray A.P., Villars-Méchin A., Malcus C., Poitevin-Later F., Lepape A., Monneret G. (2012). IL-7 restores lymphocyte functions in septic patients. J. Immunol..

[72] Ferrari-Lacraz S., Ferrari S. (2011). Do RANKL inhibitors (denosumab) affect inflammation and immunity?. Osteoporos. Int..

[73] Dougall W.C., Glaccum M., Charrier K., Rohrbach K., Brasel K., de Smedt T., Daro E., Smith J., Tometsko M.E., Maliszewski C.R. (1999). RANK is essential for osteoclast and lymph node development. Genes Dev..

[74] Kayagaki N., Yamaguchi N., Abe M., Hirose S., Shirai T., Okumura K., Yagita H. (2002). Suppression of antibody production by TNF-related apoptosis-inducing ligand (TRAIL). Cell. Immunol..

[75] Kim D., Mebius R.E., MacMicking J.D., Jung S., Cupedo T., Castellanos Y., Rho J., Wong B.R., Josien R., Kim N. (2000). Regulation of peripheral lymph node genesis by the tumor necrosis factor family member TRANCE. J. Exp. Med..

[76] Yun T.J., Tallquist M.D., Aicher A., Rafferty K.L., Marshall A.J., Moon J.J., Ewings M.E., Mohaupt M., Herring S.W., Clark E.A. (2001). Osteoprotegerin, a crucial regulator of bone metabolism, also regulates B cell development and function. J. Immunol..

[77] Cummings S.R., San Martin J., McClung M.R., Siris E.S., Eastell R., Reid I.R., Delmas P., Zoog H.B., Austin M., Wang A. (2009). Denosumab for prevention of fractures in postmenopausal women with osteoporosis. N. Engl. J. Med..

[78] Anastasilakis A.D., Toulis K.A., Goulis D.G., Polyzos S.A., Delaroudis S., Giomisi A., Terpos E. (2009). Efficacy and safety of denosumab in postmenopausal women with osteopenia or osteoporosis: A systematic review and a meta-analysis. Horm. Metab. Res..

[79] Ellis G.K., Bone H.G., Chlebowski R., Paul D., Spadafora S., Smith J., Fan M., Jun S. (2008). Randomized trial of denosumab in patients receiving adjuvant aromatase inhibitors for nonmetastatic breast cancer. J. Clin. Oncol..

[80] Watts N.B., Roux C., Modlin J.F., Brown J.P., Daniels A., Jackson S., Smith S., Zack D.J., Zhou L., Grauer A. (2012). Infections in postmenopausal women with osteoporosis treated with denosumab or placebo: Coincidence or causal association?. Osteoporos. Int..

[81] Toulis K.A., Anastasilakis A.D. (2010). Increased risk of serious infections in women with osteopenia or osteoporosis treated with denosumab. Osteoporos. Int..

[82] Gong L., Altman R.B., Klein T.E. (2011). Bisphosphonates pathway. Pharmacogenet. Genom..

[83] Shikama Y., Nagai Y., Okada S., Oizumi T., Shimauchi H., Sugawara S., Endo Y. (2010). Pro-IL-1β accumulation in macrophages by alendronate and its prevention by clodronate. Toxicol. Lett..

[84] Frith J.C., Mönkkönen J., Blackburn G.M., Russell R.G., Rogers M.J. (1997). Clodronate and liposome-encapsulated clodronate are metabolized to a toxic ATP analog, adenosine 5′-(beta, gamma-dichloromethylene) triphosphate, by mammalian cells in vitro. J. Bone Miner. Res..

[85] Lehenkari P.P., Kellinsalmi M., Näpänkangas J.P., Ylitalo K.V., Mönkkönen J., Rogers M.J., Azhayev A., Väänänen H.K., Hassinen I.E. (2002). Further insight into mechanism of action of clodronate: Inhibition of mitochondrial ADP/ATP translocase by a nonhydrolyzable, adenine-containing metabolite. Mol. Pharmacol..

[86] Drake M.T., Clarke B.L., Khosla S. (2008). Bisphosphonates: Mechanism of action and role in clinical practice. Mayo Clin. Proc..

[87] Schindeler A., Little D.G. (2007). Bisphosphonate action: Revelations and deceptions from in vitro studies. J. Pharm. Sci..

[88] Reszka A.A., Rodan G.A. (2003). Bisphosphonate mechanism of action. Curr. Rheumatol. Rep..

[89] Rodan G.A., Reszka A.A. (2002). Bisphosphonate mechanism of action. Curr. Mol. Med..

[90] Luckman S.P., Hughes D.E., Coxon F.P., Graham R., Russell G., Rogers M.J. (1998). Nitrogen-containing bisphosphonates inhibit the mevalonate pathway and prevent post-translational prenylation of GTP-binding proteins, including Ras. J. Bone Miner. Res..

[91] Dunford J.E., Thompson K., Coxon F.P., Luckman S.P., Hahn F.M., Poulter C.D., Ebetino F.H., Rogers M.J. (2001). Structure-activity relationships for inhibition of farnesyl diphosphate synthase in vitro and inhibition of bone resorption in vivo by nitrogen-containing bisphosphonates. J. Pharmacol. Exp. Ther..

[92] Luckman S.P., Coxon F.P., Ebetino F.H., Russell R.G., Rogers M.J. (1998). Heterocycle-containing bisphosphonates cause apoptosis and inhibit bone resorption by preventing protein prenylation: Evidence from structure-activity relationships in J774 macrophages. J. Bone Miner. Res..

[93] Coxon F.P., Rogers M.J. (2003). The role of prenylated small GTP-binding proteins in the regulation of osteoclast function. Calcif. Tissue Int..

[94] Coxon F.P., Helfrich M.H., Van’t Hof R., Sebti S., Ralston S.H., Hamilton A., Rogers M.J. (2000). Protein geranylgeranylation is required for osteoclast formation, function, and survival: Inhibition by bisphosphonates and GGTI-298. J. Bone Miner. Res..

[95] Deng X., Tamai R., Endo Y., Kiyoura Y. (2009). Alendronate augments interleukin-1beta release from macrophages infected with periodontal pathogenic bacteria through activation of caspase-1. Toxicol. Appl. Pharmacol..

[96] Deng X., Yu Z., Funayama H., Shoji N., Sasano T., Iwakura Y., Sugawara S., Endo Y. (2006). Mutual augmentation of the induction of the histamine-forming enzyme, histidine decarboxylase, between alendronate and immuno-stimulants (IL-1, TNF, and LPS), and its prevention by clodronate. Toxicol. Appl. Pharmacol..

[97] Schneider E., Rolli-Derkinderen M., Arock M., Dy M. (2002). Trends in histamine research: New functions during immune responses and hematopoiesis. Trends Immunol..

[98] Miyagawa F., Tanaka Y., Yamashita S., Minato N. (2001). Essential requirement of antigen presentation by monocyte lineage cells for the activation of primary human gamma delta T cells by aminobisphosphonate antigen. J. Immunol..

[99] Kunzmann V., Bauer E., Feurle J., Weissinger F., Tony H.P., Wilhelm M. (2000). Stimulation of gammadelta T cells by aminobisphosphonates and induction of antiplasma cell activity in multiple myeloma. Blood.

[100] Thompson K., Rogers M.J. (2004). Statins prevent bisphosphonate-induced gamma, delta-T-cell proliferation and activation in vitro. J. Bone Miner. Res..

[101] Masuda T., Deng X., Tamai R. (2009). Mouse macrophages primed with alendronate down-regulate monocyte chemoattractant protein-1 (MCP-1) and macrophage inflammatory protein-1alpha (MIP-1alpha) production in response to Toll-like receptor (TLR) 2 and TLR4 agonist via Smad3 activation. Int. Immunopharmacol..

[102] Li X., Qin L., Bergenstock M., Bevelock L.M., Novack D.V., Partridge N.C. (2007). Parathyroid hormone stimulates osteoblastic expression of MCP-1 to recruit and increase the fusion of pre/osteoclasts. J. Biol. Chem..

[103] Choi S.J., Cruz J.C., Craig F., Chung H., Devlin R.D., Roodman G.D., Alsina M. (2000). Macrophage inflammatory protein 1-alpha is a potential osteoclast stimulatory factor in multiple myeloma. Blood.

[104] Ganguli A., Steward C., Butler S.L., Philips G.J., Meikle S.T., Lloyd A.W., Grant M.H. (2005). Bacterial adhesion to bisphosphonate coated hydroxyapatite. J. Mater. Sci. Mater. Med..

[105] Thompson K., Freitag L., Styger U., Camenisch K., Zeiter S., Arens D., Richards R.G., Moriarty T.F., Stadelmann V.A. (2020). Impact of low bone mass and antiresorptive therapy on antibiotic efficacy in a rat model of orthopedic device-related infection. J. Orthop. Res..

[106] Li H., Hong S., Qian J., Zheng Y., Yang J., Yi Q. (2010). Cross talk between the bone and immune systems: Osteoclasts function as antigen-presenting cells and activate CD4+ and CD8+ T cells. Blood.

[107] Thillemann T.M., Pedersen A.B., Mehnert F., Johnsen S.P., Søballe K. (2010). Postoperative use of bisphosphonates and risk of revision after primary total hip arthroplasty: A nationwide population-based study. Bone.

[108] Li D., Gromov K., Proulx S.T., Xie C., Li J., Crane D.P., Søballe K., O’Keefe R.J., Awad H.A., Xing L. (2010). Effects of antiresorptive agents on osteomyelitis: Novel insights into the pathogenesis of osteonecrosis of the jaw. Ann. N. Y. Acad. Sci..

[109] Yamashita J., Sawa N., Sawa Y., Miyazono S. (2021). Effect of bisphosphonates on healing of tooth extraction wounds in infectious osteomyelitis of the jaw. Bone.

